# Suicidal ideation in the context of alcohol use among college students: differences across sexual orientation and gender identity

**DOI:** 10.1007/s00127-024-02736-9

**Published:** 2024-07-30

**Authors:** John K. Kellerman, Shireen L. Rizvi, Paul R. Duberstein, Evan M. Kleiman

**Affiliations:** Rutgers, The State University of New Jersey, 629 Tillett Hall 53 Avenue E, Piscataway, 08854 NJ US

**Keywords:** Alcohol, Suicidal ideation, College Students, Sexual/Gender minority

## Abstract

**Purpose:**

There is a dearth of research on suicidal ideation (SI) that occurs specifically in the context of drinking alcohol. Alcohol use and binge drinking are both elevated among college students, among whom sexual and gender minority (SGM) students are at particular risk for SI. This manuscript examines alcohol use, SI, and SI specifically in the context of alcohol use among a large sample of undergraduate students and examines differences across sexual and gender minority groups.

**Methods:**

Data were drawn from ~ 300,000 students who completed the American College Health Association National College Health Assessment (ACHA-NCHA) between Spring 2019 and Fall 2022. Participants reported identity variables and information about drinking behaviors and suicidal ideation over the past year. Multilevel models were used for all analyses.

**Results:**

Risky drinking behaviors and higher blood alcohol content during the last episode of social drinking were associated with higher odds of SI while drinking. Rates of risky drinking behaviors, SI, and SI while drinking were elevated among SGM students with SGM men and nonbinary students reporting the highest rates across groups.

**Conclusion:**

SI while drinking, which is seldom assessed in measures of either measures of suicidal thoughts or alcohol use behavior, is an important construct for further research to improve our understanding of high risk states for suicide. Given elevated rates of alcohol use and SI among college students, providing education and resources to reduce SI while drinking is a critical target for universities, particularly to reduce risk among vulnerable SGM students.

## Introduction

### Alcohol consumption among college students

Early adulthood represents a period of high alcohol consumption. Multiple studies have found that drinking behavior increases during late adolescence and reaches a lifetime peak in the 20s [1–3]. Drinking behavior is further elevated in college environments, with college students demonstrating disproportionately frequent alcohol consumption and higher binge drinking rates compared to same-age peers who do not attend college [4]. Although estimates vary by source, approximately 67% of college students report drinking any alcohol within the past 30 days and approximately 38% report binge drinking, defined by the Substance Abuse and Mental Health Services Administration as consumption of 5 or more drinks in one occasion [5, 6]. Frequent alcohol consumption and binge drinking are associated with numerous social and health consequences, including impaired academic performance, injuries, drunk driving, and interpersonal problems [7–9]. Drinking episodes where individuals have higher blood alcohol concentrations (BACs) are associated with increased likelihood of negative outcomes, likely due to higher degrees of impairment across multiple domains of psychomotor and cognitive performance, including driving performance, memory, divided attention, and increased risk-taking [6, 10–12].

### Group differences in alcohol use

Among both adults and adolescents, compared to women, men drink alcohol significantly more often and in higher quantities [9]. Men also experience significantly higher rates of risky behaviors, injury, and death in the context of alcohol use, due in part to drinking more heavily on average than women [13, 14]. Historically, this gender disparity in alcohol use was consistent among college students [15, 16]. More recent large-scale studies have found that the gender gap in drinking among college students has reduced and in some cases disappeared, with women now demonstrating drinking behavior comparable to men [17, 18].

Sexual and gender minority (SGM) adults and adolescents report higher rates of both alcohol consumption and binge drinking behavior compared to their cisgender and heterosexual peers, a difference that recent studies have also found among college students [19–23]. Greater disparities in alcohol use have been observed between sexual minority women and cisgender/heterosexual women compared to men, suggesting an interaction effect between gender and sexual orientation in the occurrence of heavy drinking [23, 24]. Research on gender minority individuals remains limited, although preliminary work has found higher frequency of binge drinking behavior and higher scores on the Alcohol Use Disorders Identification Test (AUDIT), a test that assesses both drinking behaviors and alcohol-related problems, among gender minority people compared to cisgender peers [25–27]. Research that examines subgroup differences among gender minority participants (e.g., trans women, trans men, nonbinary individuals) is even more sparse, limiting the ability to characterize alcohol consumption among heterogenous groups and to identify which individuals may be important targets for education and intervention [28].

### Co-occurring alcohol consumption and suicidal thoughts and behavior

Risky drinking behaviors are associated with elevated mental health difficulties among college students, including anxious and depressive symptoms [29–31] and suicidal thoughts and behaviors (STBs) [32–34]. Indeed, college students who reported binge drinking even one time in the previous year were over twice as likely to report seriously considering suicide [35]. The majority of research examining the relationship between suicide and alcohol consumption has been conducted using cross-sectional methods or longitudinal designs conducted over periods of months or years. These studies have identified distal relationships between overall alcohol consumption behavior and suicide risk, but less work that has examined alcohol consumption as an acute risk factor for STBs. There is a further dearth of research investigating the occurrence of STBs *while* drinking. The few studies that have examined this phenomenon have focused specifically on suicidal behavior and have found that a substantial percentage of suicide attempts and deaths take place in the context of alcohol use [36–39]. A 2004 review of suicide attempt and suicide death studies found that approximately 40% of individuals who attempted or died by suicide had consumed alcohol within the few hours prior to the attempt while other studies identified even higher rates of positive blood alcohol concentrations among suicide attempters and those who die by suicide [36–39]. There is nearly no research, however, on suicidal *ideation (SI)*, a key risk factor for suicidal behavior, in the context of acute alcohol use. Recent research indicates that SI may be more intense on days when alcohol is consumed and qualitative data suggests that SI that occurs while drinking is a complex phenomenon, influenced by multiple factors including mood and quantity of alcohol consumed [40]. Researchers have called for investigations into the occurrence of suicidal thoughts while engaging in various degrees of alcohol consumption to inform efforts to reduce alcohol-involved suicidal behavior [41]. The present paper aims to address this crucial gap and to describe patterns of SI in the context of alcohol use among college students.

### Group differences in suicidal thoughts and behaviors

Similarly to alcohol use differences, SGM college students report higher rates of suicidal thoughts and behaviors compared to cisgender and heterosexual college students [42]. Identity-related minority stressors, including harassment, discrimination, and social rejection are associated with higher rates of suicidal thoughts and behaviors among SGM adolescents and young adults, and direct experiences of discrimination and victimization on the basis of identity specifically predict higher rates of suicidal thoughts and suicide attempts among SGM college students [43–45]. However, the extent of the disparity in suicide risk observed among SGM individuals is only partially explained by traditional risk factors (e.g., depression) and by minority stressors, suggesting that the identification of additional risk factors may be critical to better understand the elevated suicide rate. Given elevated rates of alcohol use and SI among SGM college students, we anticipated that suicidal thoughts in the context of drinking would be more prevalent among SGM students, potentially elucidating a risk factor for suicide that is particularly relevant to understand and ultimately reduce suicide risk among a vulnerable population. Accordingly, the present study examines alcohol use, SI, and suicidal ideation specifically in the context of alcohol use among a large sample of students and examines differences across sexual and gender minority groups.

## Method

### Participants

This study utilizes data from the American College Health Association National College Health Assessment (ACHA-NCHA), a largescale, national survey that since 2000 has collected data from nearly 2 million undergraduate and graduate students across over 1,000 institutions. Present analyses include data from Wave 3 of the survey (ACHA-NCHA III) collected between Fall 2019 and Spring 2022. The self-report survey is administered by each participating institution, with multiple recruitment and administration strategies employed across institutions. Present analyses include data from the 297,136 students in ACHA-NCHA III (see Table 1 for demographic information).

### Measures

#### Sexual orientation and gender identity

Participants were asked to report which terms they use to describe their sexual orientation and gender identity. The response options for sexual orientation were straight/heterosexual, bisexual, gay, lesbian, pansexual, queer, and questioning. Responses were dichotomized into heterosexual and sexual minority (any response other than “straight/heterosexual”). The response options for gender identity were woman or female, man or male, trans woman, trans man, genderqueer, agender, genderfluid, intersex, and non-binary. A separate question asked “do you identify as transgender” with dichotomous response options (i.e., Yes/No). Participants who reported “yes” to whether they identified as transgender OR who reported a gender identity other than “man or male” or “woman or female” were coded as gender minority participants.

These data were used to divide participants into 5 groups: cisgender/heterosexual men, cisgender/heterosexual women, SGM women (i.e., cisgender women who reported a sexual minority identity or transgender women), SGM men (i.e., cisgender men who reported a sexual minority identity or transgender men), and gender expansive individuals (i.e., participants who did not report a binary gender identity, including genderqueer, agender, genderfluid, intersex, and nonbinary participants).

#### Past-year suicidal ideation

Participants were asked: “How often have you thought about killing yourself in the past year?” with response options of: “Never,” “Rarely – 1 time,” “Sometimes – 2 times,” “Often – 3–4 times,” and “Very often – 5 or more times.” For present analyses, responses were dichotomized based on whether participants had experienced suicidal ideation in the past year (i.e., endorsed any response option other than “never”).

#### Past-year suicidal ideation while drinking alcohol

Participants were asked to endorse whether they had experienced a series of adverse events while drinking alcohol. Present analyses include responses to the item: “Within the last 12 months, have you experienced any of the following while drinking alcohol? Seriously considered suicide.” Response options were dichotomous (i.e., Yes/No).

#### Risky drinking behaviors

The level and impact of individuals’ alcohol consumption was assessed using the alcohol items from the Alcohol, Smoking, and Substance Involvement Screening Test (ASSIST), a measure developed by the World Health Organization [46]. An overall risk score for alcohol use was calculated by summing scores across six questions: 1) “How often have you used alcohol?” 2) “How often have you had a strong desire or urge to use alcohol?” 3) “How often has your use of alcohol led to health, social, legal, or financial problems?” 4) How often have you failed to do what was normally expected of you because of your use of alcohol?” 5) “Has a friend or relative or anyone else ever expressed concern about your use of alcohol?” and 6) “Have you ever tried and failed to control, cut down, or stop using alcohol?” The first four questions asked about experiences over the 3 months prior to survey administration. Response options included: Never; Once or twice; Monthly; Weekly; and Daily or almost daily. The final two questions asked about experiences across participants’ entire lifetimes. Response options included: No, never; Yes, in the past 3 months; and Yes, but not in the past 3 months.

#### Blood alcohol content

The ACHA-NCHA data includes an estimated blood alcohol concentration (BAC) during the last time each participant drank socially, considering the average rate of ethanol metabolism. This was calculated using the data on participant sex assigned at birth (male, female, or intersex), height, weight, the number of drinks they consumed during the last time they drank socially or at a party, and the length of time over which those drinks were consumed.

### Analytic strategy

Given the nested structure of the data (individuals within schools), we used multilevel models with school used as a clustering variable. Analyses consisted of three multilevel models: (1) Model 1 examined the relationship between identity-based groups and risky drinking behavior to explore whether sexual orientation and gender identity predicted differences in risky behavior rates; (2) Model 2 examined the relationship between identity-based groups and past-year suicidal ideation; (3) Model 3 was constrained to participants who reported experiencing past-year suicidal ideation. This model examined the relationship between identity-based groups and suicidal ideation while drinking alcohol, with risky drinking behaviors and BAC during participants’ last experience drinking socially entered as covariates. Given school-to-school environmental differences (e.g., alcohol accessibility, campus drinking culture, student car ownership), risky drinking behaviors scores were group centered to examine whether individuals reporting higher or lower than average risky behaviors in comparison to other students within their school were more likely to experience SI while intoxicated. School type (i.e., 2-year vs. 4 or more years) was entered as a cluster-level covariate in all models. For all models that included group-level comparisons, cisgender and heterosexual men were entered as the comparison group given that this group reported the lowest overall levels of past-year suicidal ideation. The first model was a linear model whereas the second and third models were logistic models. All multilevel models were conducted using the using the lme4 R package and tables were created using the sjPlot R package.

## Results

### Descriptives

Across the sample, 229,283 (77%) participants reported being cisgender and heterosexual and 71,900 participants (23%) reported a sexual and/or gender minority identity (SGM; see Table 1 for full participant demographics). Groups were then stratified into 5 categories: (1) cisgender/heterosexual men (*N* = 79,307); (2) cisgender/heterosexual women (*N* = 146,970); (3) SGM men (*N* = 14,596); (4) SGM women (*N* = 47,259); and (5) nonbinary/gender expansive individuals (*N* = 8,430).

**Table 1:** Demographic characteristics

Sample size	297,136
Age (years)	23.07 (6.58)
Class year	
1st year	59,754 (20.1%)
2nd year	51,395 (17.3%)
3rd year	56,969 (19.2%)
4th year	45,665 (15.4%)
5th year	12, 645 (4.3%)
Master’s student	37, 955 (12.8%)
Doctoral student	29,889 (10.1%)
Other	3,070 (1.0%)
Sexual Orientation	
Straight/Heterosexual	227,515 (77.2%)
Bisexual/Pansexual	39,102 (13.2%)
Gay/Lesbian/Homosexual	12,166 (4.2%)
Queer	6,170 (2.1%)
Questioning	7,542 (2.5%
Another identity	1,486 (0.5%)
Gender Identity	
Cisgender Woman	194, 124 (65.3%)
Cisgender Man	93, 090 (31.3%)
Transgender Woman	375 (0.13%)
Transgender Man	813 (0.27%)
Nonbinary	5,114 (1.72%)
Genderqueer	1,320 (0.44%)
Agender	688 (0.23%)
Genderfluid	1,280 (0.43%)
Another identity	1,234 (0.42%)
Race/Ethnicity	
American Indian/Alaska Native	6,241 (2.1%)
Asian	48,608 (16.4%)
Black	18,800 (6.3%)
Hispanic/Latinx	44,900 (15.1%)
Middle Eastern/North African	5,303 (1.8%)
Native Hawaiian/Pacific Islander	1,820 (0.61%)
White	192,682 (64.8%)
Biracial/multiracial	13,916 (4.7%)
Another identity	4,188 (0.14%)
US Geographical Areas	
Northeast	67,377 (22.7%)
Midwest	67,296 (22.6%)
South	82,872 (27.9%)
West	83,638 (28.1%)

Note: Race/Ethnicity categories add up to greater than 100% due to option to select multiple

### Alcohol risk

Multilevel modeling results for the relationship between identity-based groups and risky drinking behavior are shown in Table 2. Nonbinary individuals’ risky drinking behavior did not significantly differ from that of cisgender/heterosexual men, and both of these groups reported higher rates of risky drinking behavior compared to cisgender/heterosexual women. Across all groups, SGM men reported the highest level of risk alcohol behavior, followed by SGM women. An additional multilevel model entering SGM status as the predictor found that SGM identity was associated with higher risky drinking behavior compared to cisgender/heterosexual individuals, regardless of gender identity (*B* = 0.51, *95% CI* = 0.45, 0.57, *p* < .001). In both models, institution type was significantly associated with elevated risky drinking behavior such that students at 4 or more year institutions reported riskier drinking behavior than students at 2-year institutions.

**Table 2:** Multilevel model showing differences in risky drinking behavior by identity group

	Risky Drinking Behavior Score		
*Predictors*	*Estimates*	*CI*	*p*
(Intercept)	6.38	5.96–6.79	**< 0.001**
Cisgender/Heterosexual Women	-0.38	-0.44 – -0.31	**< 0.001**
SGM Men	0.54	0.42–0.66	**< 0.001**
SGM Women	0.23	0.15–0.31	**< 0.001**
Nonbinary/Gender Expansive individuals	-0.03	-0.19–0.12	0.666
Four or more year institution	0.48	0.06–0.90	**0.025**
**Random Effects**			
σ^2^	33.61		
τ_00 PERMID_	0.56		
ICC	0.02		
N _PERMID_	373		
Observations	206,319		
Marginal R^2^ / Conditional R^2^	0.003 / 0.019		
Deviance	1311398.447		
log-Likelihood	-655710.644		

Note: Cisgender/heterosexual men were entered as the comparison group

### Suicidal ideation

Across the sample, 31.2% of participants (*N* = 93,063) reported some level of suicidal ideation over the 12 months prior to survey administration. Among participants who reported SI in the past 12 months, 39.6% (*N* = 36,879) reported a SGM identity.

Multilevel modeling results for the relationship between identity-based groups, risky drinking behavior, and past-year suicidal ideation are shown in Table 3. Results indicated that cisgender/heterosexual men were the least likely to report experiencing SI in the past 12 months. The confidence intervals between each identity group did not overlap with each other, indicating that the groups significantly differed from each other. In comparison to cisgender/heterosexual men, nonbinary individuals exhibited the highest likelihood of experiencing SI, followed by SGM women, SGM men, and cisgender/heterosexual women in that order. Institution type was also significantly associated with past-year SI, with students at 2-year institutions reporting higher rates of SI than students at 4 or more year institutions.

**Table 3:** Logistic multilevel model showing differences in prevalence of suicidal ideation over the past 12 months by identity group

	Suicidal Ideation		
*Predictors*	*Odds Ratios*	*CI*	*p*
(Intercept)	0.39	0.35–0.43	**< 0.001**
Cisgender/Heterosexual Women	1.04	1.02–1.06	**< 0.001**
SGM Men	2.46	2.37–2.55	**< 0.001**
SGM Women	3.21	3.13–3.29	**< 0.001**
Nonbinary/Gender Expansive individuals	5.64	5.38–5.92	**< 0.001**
Four or more year institution	0.86	0.77–0.95	**0.003**
**Random Effects**			
σ^2^	3.29		
τ_00 PERMID_	0.03		
ICC	0.01		
N _PERMID_	373		
Observations	295,755		
Marginal R^2^ / Conditional R^2^	0.071 / 0.080		
Deviance	347913.698		
log-Likelihood	-173956.849		

Note: Cisgender/heterosexual men were entered as the comparison group

### Suicidal ideation while drinking alcohol

The final multilevel model examined differences in suicidal ideation while drinking alcohol across identity groups. Both higher rates of risky drinking behavior and higher BAC during the last episode of drinking were associated with higher odds of suicidal ideation while drinking alcohol over the past 12 months. Among both cisgender/heterosexual and SGM individuals, men experienced higher odds of SI while drinking alcohol compared to women. All SGM groups reported higher likelihood of SI while drinking alcohol compared to cisgender/heterosexual participants, with nonbinary individuals demonstrating the highest risk. Institution type did not predict likelihood of experiencing SI while drinking alcohol.

**Table 4:** Logistic multilevel model showing the relationship between identity groups, risky drinking behaviors, blood alcohol content during the last episode of drinking, and odds of suicidal ideation while drinking alcohol in the past year

	Suicidal Ideation while Drinking		
*Predictors*	*Odds Ratios*	*CI*	*p*
(Intercept)	0.06	0.05–0.08	**< 0.001**
Cisgender/Heterosexual Women	0.68	0.62–0.75	**< 0.001**
SGM Men	1.50	1.32–1.70	**< 0.001**
SGM Women	1.18	1.08–1.29	**< 0.001**
Nonbinary/Gender Expansive individuals	1.60	1.39–1.83	**< 0.001**
Risky Drinking Behavior Score	1.11	1.10–1.11	**< 0.001**
Blood Alcohol Content during last episode of drinking	5.89	4.06–8.55	**< 0.001**
Four or more year institution	0.85	0.67–1.06	0.145
**Random Effects**			
σ^2^	3.29		
τ_00 PERMID_	0.03		
ICC	0.01		
N _PERMID_	373		
Observations	59,477		
Marginal R^2^ / Conditional R^2^	0.159 / 0.165		

Note: Cisgender/heterosexual men were entered as the comparison group

## Discussion

Findings point to important gender differences in risky drinking behavior, suicidal ideation, and suicidal ideation specifically in the context of alcohol use. Previous literature has consistently found that, on average, men engage in higher rates of risky alcohol use behaviors (e.g., property damage, violence and arrest, behavior that causes physical injury) than do women [1, 13, 14]. Present results support these gender differences among college students as well as previous work that SGM individuals have higher risky alcohol use rates than do cisgender/heterosexual individuals with SGM women reporting higher rates in the present sample than cisgender/heterosexual men. Literature on risky drinking behaviors is more limited among nonbinary and gender expansive people, especially research examining differences between nonbinary and binary samples. However, the few studies that have examined risky drinking rates among gender minority college students in general have found elevated rates compared to cisgender individuals [20, 28, 47]. Present data found that nonbinary people have similar rates of risky alcohol use to cisgender/heterosexual men but lower rates compared to binary SGM individuals. Additional research is needed to replicate this gender difference and to examine potentially unique contributors, contexts, and purposes for risky alcohol behavior among nonbinary individuals.

SGM participants reported substantially higher rates of suicidal ideation over the past year compared to cisgender and heterosexual individuals. Although only 23% of the overall sample reported a minoritized sexual orientation or gender identity, SGM participants accounted for approximately 40% of cases of reported SI. Additionally, SGM participants were almost twice as likely to experience SI, with 25.1% of cisgender/heterosexual individuals reporting some level of suicidal ideation over the past year compared to 50.5% of SGM individuals. These findings align with extensive research that have found higher rates of suicidal thoughts and behaviors among SGM populations across the lifespan [42, 48]. Suicidal ideation was higher among all SGM groups regardless of gender compared to cisgender/heterosexual participants, with nonbinary participants reporting the highest rates of SI of all groups. Although men are more likely to die by suicide, a wealth of research has found that women are more likely to experience suicidal ideation, a disparity that is born out across both cisgender/heterosexual and SGM men and women in the current sample [49–51].

Despite higher overall SI, cisgender/heterosexual women were less likely to report experiencing SI while drinking compared to cisgender/heterosexual men. This disparity persisted despite controlling for the effects of risky drinking behavior and BAC during last episode of drinking, both of which are higher among cisgender/heterosexual men and are associated with SI while drinking. Previous work has yielded mixed findings on gender differences in suicidal behavior (e.g., attempts, deaths) in the context of alcohol use. While some reviews have found that suicidal behaviors while drunk are more common among men than women, other studies indicate that alcohol use acutely raises the risk of suicide attempt more for women than for men [43, 52, 53]. This is the first study that we are aware of to observe gender differences in suicidal ideation while drinking, although more research with broader populations is needed to examine whether this trend generalizes beyond college students. This study did not assess the context in which alcohol use occurred, which may be a key factor in understanding gender differences in SI while drunk. The majority of drinking behaviors reported by college students occur in social contexts with a minority of young adults reporting that they have ever consumed alcohol while alone [54, 55]. Further evidence has found that individuals who drink for the purpose of social facilitation are significantly less likely to meet criteria for depression than those who drink for other reasons, including coping with emotional pain [54]. It is possible that factors such as social context during an episode of drinking and reasons for drinking may help to explain the gender disparity in SI while drinking. Further investigation is needed to examine factors that make men particularly vulnerable to SI specifically while drunk that may not be accounted for in studies of SI more broadly that have found inverse trends across men and women.

Similar trends were observed among SGM men compared to SGM women, with SGM men having higher rates of SI while drinking despite lower rates of SI. Nonbinary people had similar rates to SGM men and higher rates than SGM women, indicating nonbinary individuals are at particularly high risk of both SI and SI while drinking compared to other groups. Overall, all SGM groups had higher likelihood of past year SI while drinking compared to cisgender/heterosexual individuals.

Across the sample, both risky drinking behavior and BAC during previous episode of drinking socially were significant predictors of experiencing SI while drinking during the past year. These findings align with recent qualitative work suggesting that consuming larger quantities of alcohol is more likely to lead to negative mood and SI [40]. However, the percentage of students across the sample who reported SI while drinking was substantially less than both the total percentage who reported risky drinking behavior and the total percentage who reported past year SI across all contexts. Examination of the specific mechanisms and risk factors that lead to SI while drinking is essential to better understand what types of interventions might be effective to reduce both SI while drinking and the escalation from SI to suicidal behavior during a period of increased impulsivity due to alcohol consumption.

### Strengths and limitations

This study had several strengths and limitations. The dataset consisted of responses from a large sample of nearly 300,000 college students from different types of institutions and regions in the United States. Multilevel models were used to examine individual students’ responses within the context of their schools to account for school-to-school environmental differences (e.g., cultural norms around drinking, accessibility of alcohol, presence of Greek life which is associated with higher alcohol consumption) [56]. These cluster-level factors, among other potentially relevant variables, were not directly assessed and future research would benefit from the inclusion of environmental, school-level variables to better contextualize suicidal ideation that occurs while drinking. Analyses were sufficiently powered to examine meaningful group differences, including differences in SI and risky drinking behavior among nonbinary students. Although some previous work has studied alcohol use and suicidal behavior [39, 53], this is one of the first studies to focus on SI while drinking and the first to do so among a college student sample who may be particularly likely to engage in risky drinking behaviors and to experience associated consequences. This paper helps to establish SI while drinking as an important construct for further research and intervention efforts to help improve safety and reduce suicide risk among a vulnerable population. This study also explores gender differences and illustrates elevated risk for SI while drinking among SGM students.

Present analyses did not include racial and ethnic identity, potentially limiting the generalizability of present findings. Previous research has found that white college students and college-aged adults are more likely to engage in heavy episodic drinking compared to students who are racially and ethnically minoritized [57–59]. Additionally, experiences of discrimination on the basis of racial/ethnic identity is associated with numerous poor mental health outcomes, including alcohol use and suicidal ideation, among racially and ethnically minoritized college students [60, 61]. These minority stress experiences may be particularly relevant and common for students who hold multiple minoritized identities (e.g., racial/ethnic minority and sexual/gender minority) [62]. Therefore, future research should examine intersectional experiences among college students to better understand how identity-related factors may lead to SI while drinking.

Although a novel construct, the single item used to assess SI while drinking is broad and does not allow for nuanced examination of the phenomenology of SI in the context of alcohol use. SI was similarly assessed using a single question and the use of longer measures would allow for more granular exploration of the nature, intensity, and frequency of suicidal thoughts across the sample. Risky drinking behavior was similarly operationalized using a single composite score of multiple types of risky behaviors. Future research would benefit from a more nuanced exploration of specific types of risky drinking behaviors and whether these differ in prevalence or in their relationship to SI while drinking across various identity groups. Data was cross-sectional and responses were retrospective across extended periods of time. Recent qualitative research suggests a bidirectional relationship between alcohol consumption and SI, such that alcohol use can lead to SI and SI can lead to alcohol use [40]. Future longitudinal research may help to establish both temporal relationships between SI, alcohol use, and SI while drinking as well as to illuminate the real-time experiences of SI while drinking and the contexts in which it occurs.

### Implications

The elevated rates of SI across the sample underlines the critical mental health issues facing college students and the need for campus resources and suicide prevention efforts to reduce both risk of suicide and the general burden associated with SI. Over 50% of SGM students reported SI in the past year - twice the rate of cisgender/heterosexual students. A recent national survey found that SGM students who had access to campus mental health services had 84% lower odds of attempting suicide while in college [63]. However, SGM students also report unique barriers to accessing and benefitting from campus mental health services [64, 65], illustrating the need for SGM-informed and affirming services on college campuses.

SI while drinking is seldom assessed in either measures of suicidal thoughts and behaviors or in measures of alcohol use behavior. Given that alcohol use is associated with increased impulsivity, a major risk factor for suicidal behavior, a clearer understanding of the phenomenology of SI while drinking is an important goal for psychological scientists and may be a useful construct to include in future research. For colleges, educating students about SI as a potential risk associated with drinking may be an important addition to existing alcohol safety instruction and programming. Explicitly connecting mental health education and resources with alcohol safety education may also help to encourage students to seek support on campus if experiencing SI while drinking.

## Data Availability

The original data set for the American College Health Association-National College Health Assessment is available by contacting the American College Health Association (https://www.acha.org//NCHA). The opinions, findings, and conclusions presented/reported in this article/presentation are those of the author(s) and are in no way meant to represent the corporate opinions, views, or policies of the American College Health Association (ACHA). ACHA does not warrant nor assume any liability or responsibility for the accuracy, completeness, or usefulness of any information presented in this article/presentation.
